# Supplementary figures and images for: The Zmat2 gene in non-mammalian vertebrates: Organizational simplicity within a divergent locus in fish
Source: PLoS One. 2020 May 28;15(5):e0233081. doi: 10.1371/journal.pone.0233081 (PMC7255616; doi:10.1371/journal.pone.0233081)

S1 Figure

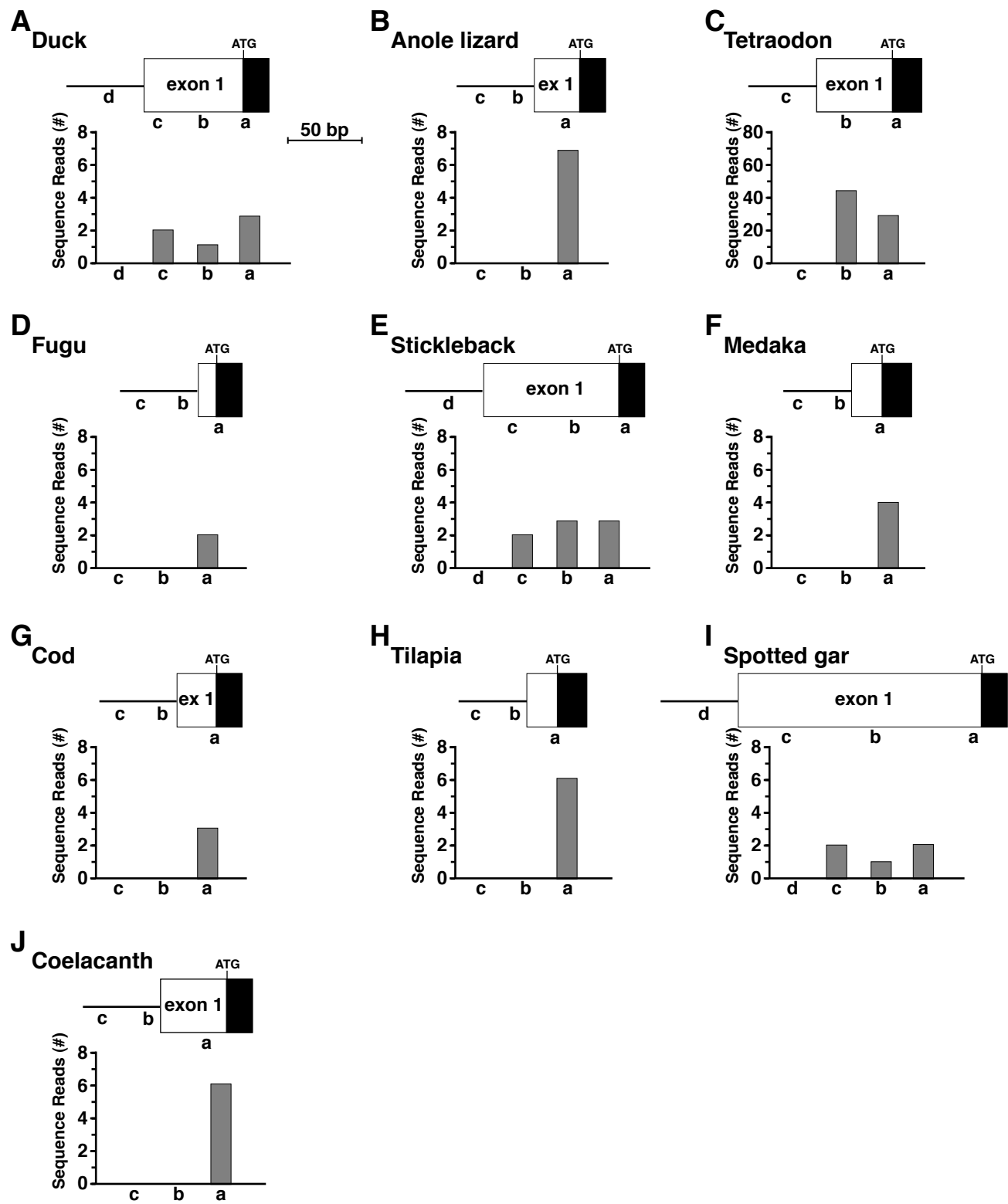

Supplement: S1 Fig — (PDF) [file pone.0233081.s001.pdf]
