# Supplementary material for: The Zmat2 gene in non-mammalian vertebrates: Organizational simplicity within a divergent locus in fish
Source: PLoS One. 2020 May 28;15(5):e0233081. doi: 10.1371/journal.pone.0233081 (PMC7255616; doi:10.1371/journal.pone.0233081)
Supplement: S1 Table — (DOCX) [file pone.0233081.s002.docx]

S1 Table: RNA-sequencing libraries screened for gene expression

| **Species** | **Tissue** | **Experiment** | **Platform** | **Layout** | **Reads sequenced**  **(x 10^6^)** |
| --- | --- | --- | --- | --- | --- |
| Anole lizard | liver | SRX2363065 | Illumina | single | 38.2 |
| chicken | liver | SRX3729588 | Illumina | paired | 64.3 |
| cod | brain | SRX1044006 | Illumina | paired | 36.5 |
| coelacanth | many | SRX112771 | Illumina | paired | 114.3 |
| duck | liver | SRX4048437 | Illumina | single | 50.4 |
| flycatcher | brain | SRX6380632 | Illumina | paired | 44.6 |
| flycatcher | liver | SRX6380621 | Illumina | paired | 53.0 |
| flycatcher | heart | SRX6380638 | Illumina | paired | 51.6 |
| flycatcher | kidney | SRX6380637 | Illumina | paired | 48.1 |
| flycatcher | testes | SRX6380636 | Illumina | paired | 58.9 |
| frog | kidney | SRX19166 | Illumina | paired | 24.1 |
| frog | heart | SRX6631057 | Illumina | paired | 62.6 |
| frog | intestine | SRX6631063 | Illumina | paired | 59.8 |
| frog | liver | SRX2704323 | Illumina | single | 58.0 |
| frog | lung | SRX6631061 | Illumina | paired | 54.1 |
| frog | muscle | SRX191168 | Illumina | paired | 34.0 |
| frog | ovary | SRX6631062 | Illumina | paired | 59.2 |
| frog | testes | SRX6631056 | Illumina | paired | 57.5 |
| fugu | spleen | SRX3871916 | Illumina | paired | 23.5 |
| medaka | brain | SRX661036 | Illumina | paired | 33.5 |
| spotted gar | brain | SRX661015 | Illumina | paired | 97.7 |
| stickleback | liver | SRX2712196 | Illumina | paired | 54.8 |
| tetraodon | brain | SRX191169 | Illumina | paired | 75.2 |
| tilapia | liver | SRX838027 | Illumina | paired | 33.1 |
| zebrafish | heart | SRX6603852 | Illumina | paired | 29.6 |
| zebrafish | liver | SRX661007 | Illumina | paired | 59.2 |
