# Supplementary material for: The Zmat2 gene in non-mammalian vertebrates: Organizational simplicity within a divergent locus in fish
Source: PLoS One. 2020 May 28;15(5):e0233081. doi: 10.1371/journal.pone.0233081 (PMC7255616; doi:10.1371/journal.pone.0233081)
Supplement: S2 Table — (DOCX) [file pone.0233081.s003.docx]

S2 Table: Probes for screening RNA-sequencing libraries

| **Gene** | **Probe** |
| --- | --- |
| **frog** |  |
| *Zmat2* ex 2-3 | GAATTACAGAAGAAAGAGAAAAGAAAGATGGCAAACCTTTACAGCCCATTAAGAGGGAAT |
| *Zmat2* ex 5-6 | TAAATCTAACTTTCCTACAATGGCACTGAATAAAGGGTATCAGTGTGTTTTGAACAAAAA |
| *Zmat2* ex 4+7 | TAAATCTAACTTTCCTACAATGGCACTGAATAAAGGGTATCAGTGTGTTTTGAACAAAAA |
| **flycatcher** |  |
| *Zmat2-1* ex 2 | AAGAACCTGGACTTCCGCCGGAAGTGGGACAAGGATGAATATGAGAAACTCGCAGAGAAG |
| *Zmat2-2* ex 2 | AAGAACCTGGACTTCCGCCGGAAATGGGACAAGGATGAATACGAGAAACTCGCAGAGAAG |
| **tilapia** |  |
| *Zmat2* ex 1-2 | ATGGCGTCAGGCAGCGGGTCGAGTAAAAACGACTTCCGACGGAAATGGGACAAAGACGAG |
| *Zmat2* ex 1-1’-2 | ATGGCGTCAGGCAGCGGGAGAGAGGAGGCAGCAGGTGGAGGTTCGAGTAAAAACGACTTC |
| *Zmat2* ex 4-5 | ACTTCCTGGATCACATCAACGGCAAAAAACAGGAACCTGGGCATGTCGATGCGGGTGGAG |
| *Zmat2* ex 4-4’-5 | CCTGGATCACATCAACGGCAAAAAACAAAATCATAGGAACCTGGGCATGTCGATGCGGGT |
